# The Evolving Regulatory Landscape for Fentanyl: China, India, and Global Drug Governance

**DOI:** 10.3390/ijerph19042074

**Published:** 2022-02-12

**Authors:** Chao Wang, Nicholas Lassi, Xiaohan Zhang, Vinay Sharma

**Affiliations:** 1 Guanghua Law School, Zhejiang University, Hangzhou 310008, China; superwang@zju.edu.cn (C.W.); Luckynickphd@gmail.com (N.L.); 2 Research Center for Socialism with Chinese Characteristics, Zhejiang University, Hangzhou 310058, China; 3 School of Law, Zhejiang University City College, Hangzhou 310015, China; 4 Academy of International Strategy and Law, Zhejiang University, Hangzhou 310008, China

**Keywords:** fentanyl, opioid epidemic, public health, China, India, regulation, rule of law, global drug governance

## Abstract

The rise of the synthetic opioid epidemic has time and time again brought criticism on China and India, the world’s two main producers of fentanyl and its chemical precursors. In the past few years, the two countries have attempted to strengthen regulations over fentanyl production and distribution, though its effects on global drug governance remain under scrutiny. This study used qualitative and comparative methods to investigate the current regulatory landscape for fentanyl, including its efficiency and potential loopholes in China and India. It concludes that although both China and India are actively and significantly attempting to step away from the global fentanyl supply chain, these efforts remain ineffective due to institutional loopholes, namely inadequate legislation and fragmented regulatory structures. From insights gained on global drug governance, we recommend a binding international convention concentrated on controlling fentanyl and its related substances, with further bilateral and multilateral cooperation among states as necessary complementation.

## 1. Introduction

First developed in 1960 by Paul Jansen, fentanyl is a potent synthetic opioid substance, primarily used for analgesia and anesthesia in the medical sector [1]. Lab made, easily modifiable, and nearly 50 to 100 times more potent than morphine, fentanyl quickly became the most used synthetic opioid in clinical settings for the management of perioperative and postoperative pain, chronic cancer pain, etc. [2]. Being highly potent and addictive, fentanyl entered the illegal drug market in 1979; it has since grown in popularity and trafficked in its pure form or in combination with other narcotics [3]. The current escalation in fentanyl availability in the U.S. is mainly a product of increased demand. As the 2000′s saw the use of prescription opioids soar, people, often addicted, gravitated to its cheaper and more potent synthetic forms. Concurrently, illegal laboratories producing fentanyl first sprang up in the U.S. in the 1990′s, they grew in number and reach, and this culminated in large-scale fentanyl production in the mid-to-late-2000′s [4,5]. U.S. domestic drug policy and the War on Drugs have been incapable of effectively controlling the fentanyl crises, thus, new global legislative regulation needs to be developed and instituted.

Its high potency resulted in an international synthetic opioid overdose crisis, with the U.S. being particularly impacted [4]. There has been an exponential increase in drug overdose deaths due to fentanyl and its analogues around the world [4,5]. Synthetic opioid (fentanyl and its related substances) overdose deaths increased by 45% in the U.S. from 2016 to 2017 [6]. The U.S. also reported a 120% rise in opioid related overdose deaths from 2010 to 2018, with 2/3rds of the deaths from synthetic opioids [7]. In Canada, synthetic opioids accounted for a population death rate of 109 per million in 2017 [8].

The U.S. Department of State reports that China has been the principal manufacturer of fentanyl trafficked to the U.S. since 2013 [9]. Chinese-manufactured fentanyl is trafficked to the U.S. through Mexico and Canada, and it’s often transported through parcel delivery services. Upon insistence from the U.S., China tightened its regulatory control of fentanyl production in 2019 [4]. This reduced China’s share of illicit global fentanyl production but caused traffickers to expand operations in neighbouring India. A 2020 DEA Intelligence Report explains, “India and China-based suspects shifted their production from China to India, likely due in part to China’s regulation of ANPP and NPP. The organization likely transferred their production to India due to difficulties obtaining precursor chemicals in China and the increasing pressure from Chinese authorities on fentanyl manufacturing operations” [10]. India has taken on a greater role in illicit fentanyl production and trafficking, both partnering with Chinese traffickers and working independently to supply the U.S. and other locations around the world [10]. All the same, inadequate regulations and their large pharmaceutical and chemical industries have positioned both China and India to remain dominate in the illicit global fentanyl network. Sino-Indian collaboration to reduce fentanyl production hasn’t quelled this serious situation.

## 2. Materials and Methods

The objective of this study is to inspire global fentanyl regulation through empirically examining the regulatory frameworks of two of the world’s largest fentanyl manufacturers, China and India. The efficacy of China’s and India’s legislative regulatory framework in the context of fentanyl production growth is analysed using comparative methods. To better understand where vulnerabilities and commonalities exist, where improvements can be made, and coalitions formed, this empirically centred comparative analysis drills down into the legislative frameworks and preventative strategies of both countries concerning fentanyl. For this purpose, we comprehensively searched domestic legislations and cases relating to fentanyl control through major legal databases including Westlaw, LexisNexis India, and Chinalawinfo, the largest database covering laws and cases from the People’s Republic of China. Related international legal sources, such as the Single Convention on Narcotic Drugs of 1961 (as amended in 1972), the Convention on Psychotropic Substances of 1971, and the United Nations Convention against Illicit Traffic in Narcotic Drugs and Psychotropic Substances of 1988, and their commentaries, are obtained by searching the United Nations Treaty Series (UNTS). Additionally, reports from a variety of regulatory, enforcement, and public health agencies, such as the National Health Commission of the People’s Republic of China (NHC), U.S. Department of State (DOS), U.S. Drug Enforcement Administration (USDEA), and the United Nations Office on Drugs and Crime (UNODC), were reviewed. These sources were selected based on their relevance, time of publication, and the depth, extent, and quality of their coverage of the fentanyl crises. Furthermore, suggestions for improving the current regulatory frameworks, both domestically and internationally, to combat fentanyl trafficking in China and India are provided.

## 3. Results

### 3.1. Fentanyl Production and Regulation in China

Along with having the world’s largest pharmaceutical and chemical industries, China has also been accused of producing the largest quantities of illicit fentanyl, accounting for 70% of its illicit global production [11]. The U.S. believes China is among the key contributors fuelling its opioid epidemic, which has grown by almost 40% from 2019 to 2020 alone [9,12]. The U.S. Department of State also reported that more than half of the global suppliers of fentanyl precursors like N-Phenethyl-4-piperidinone (NPP) and Anilino-N-phenethylpiperidine (ANPP) are based in China [9]. Drug-manufacturing equipment, such as pill presses, are also produced in China. This equipment is then shipped to places like Mexico and Canada, wherein criminal organisations use them to manufacture pills. These pills are then transported across borders into the U.S. [13]. Fentanyl is also transported through the international mailing system. In 2018, Chinese manufactured fentanyl accounted for 40% of the fentanyl seizures from international mail, most with a purity of over 90% [14].

The Chinese government first instituted regulations on fentanyl as early as 1996. The then Catalogue of Narcotic Drugs included fentanyl and eleven of its analogues under the Regulation on the Control of Narcotic Drugs and Psychotropic Drugs [15]. Remifentanil was added into the Catalogue in November of 2005, thus controlling thirteen fentanyl-related substances at that time [16]. In 2015, China included six fentanyl analogues and 110 other pharmaceutical chemicals to its list of controlled substances under its Supplementary List of Categories of Controlled Narcotics and Psychotropic Drugs for Non-medical Use [17]. These six fentanyl derivatives are acetyl-fentanyl, butyryl-fentanyl, beta-hydroxythio-fentanyl, para-fluorofentanyl, iso-butyr-fentanyl, and ocfentanyl. This Supplementary List legally banned the production, trade, trafficking, use, import, and export of fentanyl and its related substances. If medical applications of the formerly non-medical-use drugs are accepted, they can be rescheduled and catalogued under the Regulation on the Administration of Narcotic Drugs and Psychotropic Drugs. These rescheduled medical-use drugs can then be produced and sold when specially licensed [18].

In 2017, China imposed control on four more fentanyl analogues (acrylfentanyl, carfentanil, furanylfentanyl, and valerylfentanyl), and in 2018, another two fentanyl related chemicals were controlled (4-FIBF and THF-F) [19,20]. Fentanyl precursors are covered under different legislation from that of fentanyl and its analogues. Two fentanyl precursors, known as NPP and 4-ANPP, were controlled in 2018 under the Regulation on the Administration of Precursor Chemicals [21]. China further expanded its regulatory framework in 2019 by imposing class-based control on popular fentanyl analogues and substances with similar chemical structures. This class-based control was incorporated into the Supplementary Catalogue of Non-medicinal Narcotics and Psychotropic Drugs list, initiating new regulatory strategies to curb the growing number of illegal fentanyl labs in China [11]. These new measures include investigating manufacturing units, control of online trade and trafficking, increased inspection of international parcels, information sharing and collaboration with other countries, and developing technology to identify future chemicals and drugs [10]. In the span of a few years, these regulations have proven somewhat effective.

According to a 2017 report by China’s National Narcotics Control Commission, Chinese law enforcement has cracked down on the online drug market operated by Chinese drugs retailers, reportedly shutting down 1700 online vendors, arresting 21,000 people, and seizing over 60 tons of controlled narcotics and precursor substances [22]. In 2019, Chinese law enforcement reported 83,000 drug-related investigations, including 62,000 trafficking investigations and 90,000 drug related detentions. During this time, 173 manufacturing facilities were demolished, and 290 cases of illegal drug manufacturing were solved [11].

However, China’s large pharmaceutical industry and drug trafficking circles are both sensitive and counteractive to regulations governing illicit chemical substances. Though China has controlled two main fentanyl precursors, NPP and 4-ANPP, not all the precursor chemicals for fentanyl synthesis are under control. As such, China’s illicit trade network for fentanyl was transformed to market these non-scheduled precursor chemicals. Examples include Mexico’s seizure of nearly 70 kg of 4-AP, a non-scheduled precursor of ANPP, mislabelled as washing powder, and Belgium’s seizure of about 1 kg of 4,4-piperidinediol, another alternative precursor of fentanyl, both of which allegedly originated from China [23].

China’s complicated governance framework may also lead to law enforcement inefficiency. Several agencies are responsible for enforcing narcotics laws. Power and responsibility are decentralized, necessitating timely information sharing and effective cooperation among different authorities, which might fail. The National Medical Products Administration (NMPA) is the primary government authority designated to control (for medical use) narcotics and psychotropic substances. In addition to setting the annual production volume for narcotics, the NMPA is responsible for determining who is qualified to plant and maintain the raw materials, and who can distribute the drugs [24]. Other associated agencies are the Ministry of Public Security, Ministry of Agriculture, National Health Commission, National Narcotics Control Commission [22], Ministry of Transport, and the General Administration of Customs. The Ministry of Agriculture is authorized to cooperate with the NMPA to regulate the planting of raw materials. The transportation of narcotics and psychotropic substances falls under the scope of the Ministry of Transport and the General Administration of Customs. Illicit trafficking involves the Ministry of Public Security. The distribution of medical institutions and the supervision of medical practitioners falls under the National Health Commission’s surveillance. The National Narcotics Control Commission and Ministry of Public Security are responsible for the surveillance of narcotics and psychotropic substances for non-medical use [22]. As China is both a producer and transporter of fentanyl and its associated substances, enforcing regulations along border checkpoints is an important means of control. The Chinese General Administration of Customs Anti-Smuggling Bureau implements the narcotics control laws along exit points of the country, such as the seaports, airports, and land border checkpoints [25].

### 3.2. Fentanyl Production and Regulation in India

Like China, India’s pharmaceutical and chemical industries are among the largest in the world, providing extensive exports of generic pharmaceuticals. Occupying a strategic geographical location, India’s industrial strength and transportation system make it a crucial player in the global illegal drug network, both as a producer and as a transit point [10]. With China refining its regulatory framework, the global fentanyl network is shifting to the Indian pharmaceutical sector for continued production. Given that India’s pharmaceutical sector is often overlooked, and poorly regulated, Indian drug traffickers were quick to exploit China’s reduced global presence in fentanyl output. The U.S. Customs and Border Protection has acknowledged India’s rise in the global fentanyl network, and Indian drug traffickers have been prosecuted by law enforcement agencies around the world [10]. For instance, a 2018 investigation in India led to the arrest of three people in possession 10 kg of fentanyl, which was intended to be trafficked to Mexico [10].

The Narcotic Drugs and Psychotropic Substances Act (NDPS) of 1985 maintains the legal regulatory framework for controlling narcotic and psychoactive substances in India [26]. The NDPS has undergone amendments four times since its implementation, in 1988, 2001, 2014, and 2021, respectively. The 2014 amendment acknowledged the country’s need for pain relief and palliative care and introduced a list of Essential Narcotics Drugs (END). These essential pain relief drugs are controlled directly by the central government to ensure uniform countrywide access to palliative care. In 2015, the Indian government designated fentanyl and five other drugs as ENDs. With this, regulatory provisions relating to the sale, purchase, possession, consumption, and use of ENDs has been simplified. Only the State Drug Controller can permit recognised medical institutions to store and prescribe ENDs. These recognised medical institutions must submit reports and documents justifying their annual consumption of the ENDs. In 2018, the Indian government designated two direct fentanyl precursors, NPP and ANPP, as Schedule B, which restricted their export [27]. In 2020, after domestically manufactured ANPP was trafficked in Mexico, the Indian government tightened controls over ANPP and NPP by designating them as Schedule A in the NDPS Order of 2013. This order brought the domestic manufacture, distribution, sale, possession, and use of those substances under national control [23].

Additionally, the Prevention of Illicit Traffic in Narcotic Drugs and Psychotropic Substances Act was put into force in 1988. It allows for the detention of traffickers as a preventive measure. It states that any individual involved in drug trafficking can be detained by an executive order of the relevant authorities, such as the Joint Secretary of Revenue in the central government or relevant authorities at the state level [28].

Inadequate regulatory measures have driven India’s large pharmaceutical and chemical industries to fuel the global fentanyl grid through clandestine operations. Simplified regulations to increase the availability of essential narcotic drugs such as fentanyl also simplified their illicit production. Illicit fentanyl laboratories make easy use of India’s pharmaceutical infrastructure, and traffickers find ways into markets where fentanyl and its related substances are controlled. In addition, the Indian government has overlooked fentanyl analogues, which can be easily synthesized by changing the chemical structure of fentanyl. The NDPS Act permits the Indian government to enforce emergency controls over narcotics and psychotropic drugs, but this requires going through an extensive legislative framework involving multiple state and central level government agencies [26]. This is a game where the government is always a step or two behind. Furthermore, only two precursor chemicals used for fentanyl synthesis are catalogued. Alternative precursors, such as 4-AP, 1-BOC-4ANPP, 1-BOC-4-piperidone and 4,4-piperidinediol, have not been formally considered [27].

In 2018, India emerged as a source country of fentanyl and its precursors in the global fentanyl network [14]. Broadhurst et al. analysed the availability of fentanyl and its analogues on the internet and darknet over a period of 10 months [29]. For advertisements of non-pharmaceutical fentanyl and its analogues, 14.7% of the overall ads and 36.4% of the listings for the analogue 4-Fluoroisobutyr-fentanyl (4-FIBF), the highest percentage among all countries, were from Indian suppliers. Other analogues advertised to be shipped from India include Furanylfentanyl, Methoxyacetyl-fentanyl, and Orthomethyl-furanylfentanyl [30]. Furthermore, the NDPS Act does not consider narcotics related manufacturing equipment, such as pill press machines, which remain in large demand from countries like Mexico [26]. This manufacturing equipment is liable for regulation under Schedule M of the 1945 Drugs and Cosmetics Rules.

Several ministries and departments, operating under the Government of India as well as the State Governments, oversee the control of narcotics and psychotropic substances. Decentralization among various agencies produces coordination challenges, and weak law enforcement further undermines this regulation framework. The Narcotics Control Bureau (NCB) of India, supervised by the Ministry of Home Affairs, is the primary government authority responsible for combating narcotics trafficking. The Central Bureau of Narcotics (CBN) oversees licensing; authorizing the manufacture, import, and export of synthetic narcotics substances scheduled in the NDPS Act. Furthermore, the CBN is the chief authority coordinating with the International Narcotics Control Board (INCB) and foreign governments regarding the trade of scheduled substances internationally. Other associated agencies include the Directorate of Revenue Intelligence (DRI), National Investigative Agency (NIA), and the Border Security Forces (BSF) [11].

### 3.3. Comparison and Implications

This analysis of the regulatory landscape for fentanyl reveals both similarities and differences in the loopholes and inadequacies between China and India. As for the legislation landscape, both China and India have controlled two precursor chemicals used for fentanyl synthesis, NPP and 4-ANPP, and have not yet considered newly emergent alternative precursors such as 4-AP, 1-BOC-4ANPP, 1-BOC-4-Piperidone and 4,4-Piperidinediol.

China implemented class scheduling for fentanyl and all future fentanyl-like substances controlled under the Regulation on the Administration of Narcotic Drugs and Psychotropic Drugs (RANDPD) for non-medical use. In the case of medical use, fentanyl analogues can be rescheduled under RANDPD, which can be produced and sold under surveillance. In comparison, India’s fentanyl market is under loose regulations relating to the sale, purchase, possession, consumption, and use of fentanyl and its related substances. Moreover, in India, fentanyl can be more easily diverted into the illicit global market.

As for law enforcement, both China and India face collaboration challenges within their domestic operations. High legal fragmentation and pluralism in the regulatory framework, ambiguous power sharing, and the involvement of multiple state and central agencies make the regulatory framework highly ineffective. This approach to power sharing creates a large gap between resource expenditure and the benefits obtained. Insufficiently qualified human capital also leads to inefficient law enforcement. Furthermore, legislative acts extend less focus on the digital methods of distribution and procurement of fentanyl and its related substances.

#### 3.3.1. Fragmentation of Global Fentanyl Governance

Both China and India have implemented their own individual domestic laws, with different levels of regulation, on fentanyl and its related substances. Inconsistencies in domestic regulations leads to fragmentation in global fentanyl governance. This fragmentation provides an opportunity for illicit laboratories to exist and evolve with domestic law. Comparing their domestic legislation reveals similarities and differences in inadequacies and loopholes. For instance, China’s recent tightening of regulations motivated India to take on a greater role in fentanyl production. Mexico is also taking on a more prominent position in the production and supply of fentanyl. As fentanyl can be synthesized without planting raw materials, its source will continue to diversify [10].

To better control fentanyl and other catalogued substances, both China and India initiated some bilateral cooperation with countries where fentanyl is controlled. In 2020, India and the United States established a bilateral Counternarcotics Working Group to expand cooperation combating the trafficking of precursors and illicit drugs. This working group is particularly focused on synthetic opioids such as fentanyl, tramadol, and tapentadol [11]. The U.S.-China Bilateral Drug Intelligence Working Group and the Counter Narcotics Working Group provide a platform to exchange information on drug trends, discuss regulations, and improve cooperation on investigations [11]. However, there is no formal treaty that is legally binding for both sides.

To facilitate international cooperation and bridge regulatory gaps among states, the need for a coherent system of global governance of fentanyl has never been greater. The global governance of fentanyl and its related substances falls in the purview of international law, which establishes the authority and responsibility of states to maintain the health of their populations, as well as duties of international cooperation [31]. Signatory states are required to internalize the terms of international law into their domestic legal systems [32]. The three international conventions concerned with global fentanyl governance are the Single Convention on Narcotic Drugs (1961), the United Nations Convention on Psychotropic Substances (1971), and the United Nations Convention Against Illicit Traffic in Narcotic Drugs and Psychotropic Substances (1988). Both China and India are signatories of these three conventions, and both have adopted the terms of these conventions into their domestic law to varying degrees. However, the fentanyl regulation frameworks in China and India show a fragmented global fentanyl governance landscape. It reveals that existing international law cannot serve as a comprehensive and universal solution to fentanyl regulation.

#### 3.3.2. Existing Global Drug Governance Cannot Completely Cover the Fentanyl Problem

Bilateral cooperative efforts between China and India have been initiated, though, no binding bilateral treaties have been signed. Due to loopholes, the available conventions for controlling global narcotics and psychotropic substances do not completely cover the fentanyl problem. The Single Convention on Narcotic Drugs (1961) aims to schedule the medical and scientific use of drugs by implementing a four-tier schedule list [33]. Fentanyl and twenty-six fentanyl analogues are catalogued in Schedule I, subjecting them all to the applicable measures of control under this Convention. However, conventional legislative approaches are incapable of matching the rapidly increasing number of fentanyl analogues, allowing a blind spot for suppliers who can alter the chemical structure without breaching the law. The production and trafficking of these “legal substances” goes unnoticed until they have achieved significant growth or have made a significant impact. Moreover, the 1961 Convention only covers the scheduling of narcotics for medical and scientific use. Member states are uncompelled to apply the provisions of the 1961 Convention to drugs used for nonmedical or non-scientific purposes [33]. It leaves immense room for regulatory arbitrage, allowing fentanyl and its analogues to be diverted illicitly for recreational use.

In 2017, two important intermediates in the Siegfried synthetic route to produce fentanyl clandestinely, NPP and 4-ANPP, were placed in Table I (controlled drug precursors) in the United Nations Convention Against Illicit Traffic in Narcotic Drugs and Psychotropic Substances [34]. Both China and India have controlled these two substances on a national level. Though, in addition to the more common, and now highly regulated, Siegfried method, sophisticated alternative methods to produce fentanyl are gaining traction. As regulatory pressure is placed on a popular production method, others become invented, refined, or popularized. To evade the regulations placed on NPP and ANPP, innovative fentanyl laboratories utilize the Janssen method or other methods instead of the Siegfried method to produce fentanyl. The Janssen method, previously thought to be beyond the skills of most clandestine laboratory operators, involves benzylfentanyl and norfentanyl as chemical precursors instead of NPP and ANPP. Other methods can involve 4-AP as an alternative precursor chemical to NPP. Both benzylfentanyl and 4-AP are not regulated under international law and are legally available from chemical suppliers. Benzylfentanyl and 4-AP have no accepted use other than synthesizing fentanyl [10]. More and more chemical precursors are emerging without international control. In 2020, 144 fentanyl-related substances were found with no legitimate uses. 122 of these substances were not under international control and 43 were possible fentanyl precursors [11]. Placing newly emerging fentanyl-related substances under the 1961 Convention or the 1988 Convention, by gathering evidence and making a scientific review of harms, can be time-consuming [11].

Furthermore, the 1961 and 1988 Conventions are not self-executing and need to be incorporated into domestic law for their provisions to be applicable [2,33,34]. However, the extent of incorporation varies among member states. According to the International Narcotics Control Board [35], some countries legislative amendments relating to NPP and ANPP were made well after the 1988 Conventions effective date [34]. There is no enforcement authority over noncompliance among member states, thus weakening the enforceability of these international conventions.

## 4. Discussion

Given that existing domestic and international law insufficiently regulates fentanyl and its related substances, a more coherent global fentanyl governance framework needs to be established. To provide a unified and standardized code of regulation, a binding international convention concentrated on fentanyl control is necessary. This international convention can be under the United Nations system, cooperating closely with the WHO and INCB. Several factors must be taken into consideration. First, to balance the medical use of fentanyl substances and the potential for abuse and dependence, clear definitions for fentanyl analogues should be declared. Second, as fentanyl can be easily modified and synthesized using several methods with uncontrolled precursors, controlling all potential fentanyl analogues and alternative chemical precursors with similar structures is necessary. Third, a mechanism for technical and financial support for states should be implemented to facilitate compliance with the obligations under the convention. Noncompliance often results from states lacking the capacity and funding to engage these problems [36]. Technical support will increase information/intelligence sharing, and funding for advanced detection devices for fentanyl and its related substances will be advantageous for law enforcement. Fourth, an independent entity that monitors the production and flow of fentanyl substances, perhaps a fentanyl substances diversion, should be instituted. Member states are to make annual reports.

There is huge disparity among states concerning the availability and influence of fentanyl. Also, states are often unwilling to sacrifice their sovereignty to binding international law, and there have been movements within some states for the decriminalization of drugs, which may negatively influence how they interpret enforcement-based international law. Therefore, only limited commitments should be expected if a universal consensus is to be reached [31]. A single international convention will insufficiently disrupt complicated fentanyl issues. As a result, strengthening bilateral and multilateral cooperation, such as cooperation in identifying newly emerged fentanyl analogues, illicit manufacturers, and traffickers, is necessary. The aforementioned convention will provide a platform for promoting this cooperation. Considering that illicit fentanyl and its chemical precursors are also trafficked to the U.S. through Mexico and Canada and through parcel delivery services [9], closer treaty-based cooperation should be adopted among China, India, Mexico, Canada, and the U.S. in the monitoring of fentanyl trafficking [37]. Meanwhile, strengthening domestic law-based governance is also fundamental to multilateral cooperation on fentanyl regulation. For example, China, during its central conference in November of 2020, highlighted securing and advancing “the interaction between domestic rule of law and foreign-related rule of law,” which is a key element of Xi Jinping Thought on the Rule of Law, and that, “efforts shall be made to adhere to actively advancing legislation in important fields such as…public health…and foreign-related rule of law,” according to the State Council’s 2021 Legislative Work Plan [38].

Finally, the darknet is an ever-evolving platform for illicit fentanyl transactions. The international nature of the darknet appeals to the international fentanyl market. Openly accessible, anonymous, and enormous, the internet provides a relatively safe place for individuals of all ages, nationalities, and backgrounds to engage with suppliers from across the globe. This provides suppliers 24/7 accessibility, a wide reach, and high profits. At the same time, buyers get greater availability, wide ranging products, and competitive prices. Since the national regulatory authorities lack the resources and ability to detect or govern many online interactions, a “grey zone” is generated on the internet which remains outside of a regulatory context. This grey zone is a blooming marketplace for fentanyl and similar synthetic psychoactive substances [39]. Greater global cooperation is needed to govern illicit transactions in this darknet [40,41].

## 5. Conclusions

Fentanyl is easy to manufacture, highly potent, addictive, cheaply produced, and its potency-to-weight ratio provide the perfect opportunity for illicit drug suppliers in the pharmaceutical and chemical industries of China and India to enter its illicit global network. That fentanyl can be easily modified and synthesized using several methods with different chemical precursors makes it difficult to regulate. Though China and India, recognized as two main producers of fentanyl and its chemical precursors, have placed fentanyl and its related substances under regulation, both of their domestic legislations have been unable to effectively tackle this issue.

Global fentanyl governance is fragmented. Three international conventions are available for global drug governance, but they are unable to sufficiently cover the issues presented by fentanyl. New variants of fentanyl like substances are regularly springing up. They are usually unregulated and require significant time before the existing conventions consider and enact control. This commonly leaves the illicit producers and traffickers ahead of the game. Additionally, the non-executing nature of these conventions requires states to infuse these provisions into their domestic laws for applicability. States often do this at their own pace and discretion, thus manifesting nonconformity and fragmentation.

In its current state, global fentanyl legislation and regulation is insufficient. Countries without a domestic fentanyl crisis are often unconcerned about fentanyl, regulating their pharmaceutical and chemical industries, etc., which impedes the implementation of treaty obligations. Systemic corruption [42,43], cooperation concerns among different enforcement agencies [44], and congested domestic legal systems also provide serious challenges to engaging the fentanyl problem. Therefore, global fentanyl governance requires the additional strength of a binding international convention.

To obtain a unified and standardized code of regulation, and to strengthen domestic regulation, a binding international convention concentrated on controlling fentanyl and its related substances is necessary. Controlling all potential fentanyl analogues and alternative chemical precursors is necessary. Mechanisms for technical and financial support for signatory states should be instituted. Further bilateral and multilateral cooperation among states is necessary, since the influence of a single international convention will be somewhat limited.

## Data Availability

Not applicable.
